# Negative mixing enthalpy and mixing enthalpy alloying leads to interface and size effects towards superb creep resistance of nickel-based single crystalline superalloys

**DOI:** 10.1093/nsr/nwaf228

**Published:** 2025-06-09

**Authors:** Junbo Zhao, Xiaoyi Yuan, Yunsong Zhao, Zhanxin Wang, Haibo Long, Shengcheng Mao, Lihua Wang, Xiaodong Han, Ze Zhang

**Affiliations:** Beijing Key Laboratory of Microstructure and Property of Advanced Materials, College of Materials Science and Engineering, Beijing University of Technology, Beijing 100124, China; Beijing Key Laboratory of Microstructure and Property of Advanced Materials, College of Materials Science and Engineering, Beijing University of Technology, Beijing 100124, China; Science and Technology on Advanced High Temperature Structural Materials Laboratory, Beijing Institute of Aeronautical Materials, Beijing 100095, China; Beijing Key Laboratory of Microstructure and Property of Advanced Materials, College of Materials Science and Engineering, Beijing University of Technology, Beijing 100124, China; Beijing Key Laboratory of Microstructure and Property of Advanced Materials, College of Materials Science and Engineering, Beijing University of Technology, Beijing 100124, China; Beijing Key Laboratory of Microstructure and Property of Advanced Materials, College of Materials Science and Engineering, Beijing University of Technology, Beijing 100124, China; Beijing Key Laboratory of Microstructure and Property of Advanced Materials, College of Materials Science and Engineering, Beijing University of Technology, Beijing 100124, China; Beijing Key Laboratory of Microstructure and Property of Advanced Materials, College of Materials Science and Engineering, Beijing University of Technology, Beijing 100124, China; Department of Materials Science & Engineering, Southern University of Science and Technology, Shenzhen 518000, China; State Key Laboratory of Silicon Materials and Department of Materials Science and Engineering, Zhejiang University, Hangzhou 310058, China

**Keywords:** nickel-based single crystalline superalloy, creep life, mixing enthalpy alloying, negative enthalpy alloying, positive enthalpy alloying

## Abstract

Nickel (Ni)-based single crystalline superalloys are the most important high-temperature service metallic materials for aircraft engine blades. These alloys’ microstructures and mechanical properties must remain stable under high temperature and stress conditions for long periods of time. Rhenium (Re) has been the most effective alloying element, which serves as a guide to distinguish the third-generation Ni-based single crystalline superalloys. However, the Re-alloying effect seems to have been exhausted in terms of further improving superalloy creep lives, although the continuous demand for longer creep lives at high temperatures persists. This study uses the mixing enthalpy alloying route by combining negative enthalpy (N-enthalpy) alloying and positive enthalpy (P-enthalpy) alloying strategies to explore a new alloying paradigm in which the Os element is uniquely selected. The P-enthalpy effect of osmium (Os) with Ni leads to the segregation of Os elements along the γ/γ′ interface, which, in turn, yields a small-size γ′ phase and narrower γ channels by the P-enthalpy-produced interface effect and size effect. The N-enthalpy effect of Os with chromium (Cr) and other alloying elements further synchronizes the formation of local chemical ordering in the γ phase channel. These synergistic interface and size effects, together with local chemical ordering, increase the stability of the microstructure and the resistance of the dislocation movement. With these, the creep life of the Os N-enthalpy alloying superalloys approaches 1273 h under 760°C/800 MPa conditions, which is almost six times more than that of the base alloy, setting a high record among all existing metals and alloys. These findings highlight the mixing enthalpy alloying paradigm of designing new materials with excellent high-temperature performance.

## Teaser

The mixing enthalpy alloying developes new alloys and super-performance.

## INTRODUCTION

Alloying is important to improve high-temperature mechanical properties by strengthening solid solutions and minimizing atomic diffusion in metal materials [1,2]. It is also a major means for developing nickel (Ni)-based superalloys for aerospace applications, by increasing their creep resistance [3,4]. The development of high-temperature alloys began with the 80Ni-20Cr alloy in 1940 [5]; subsequently, elements such as aluminium (Al), titanium (Ti), molybdenum (Mo), tungsten (W) and tantalum (Ta) were added for synergistic alloy strengthening [6,7]. The improvement of alloying and preparation processes has considerably enhanced the high-temperature mechanical properties of superalloys [8,9]. In Ni-based single crystalline superalloys, adding rhenium (Re) is an important alloying method [10,11]. The addition of Re has also been used as a guide to distinguish the third-generation nickel-based single crystalline superalloys [12,13].

The most effective Re strengthening characteristic is known as the Re effect [14,15], which is attributed to the following. First, it was suggested that Re has a low diffusion coefficient, which increases its microstructural stability at high temperatures [16,17]. Second, Re segregates at the γ/γ′ interface [18,19], which increases the strength and stability of the interface, which also reduces the coarsening rate of the γ′ phase [20,21]. Third, Re can also react with dislocations, thus increasing the resistance to the movement of dislocations [22,23]. However, the excessive use of Re also increases the risk of forming the topologically closed-packed (TCP) phase [24,25], which reduces the mechanical properties of superalloys [26,27]. Although the addition of ruthenium (Ru) can inhibit or delay the precipitation of the TCP phase [28,29], it introduces other harmful effects [30–35], which further limits the performance and engineering application of Ru-containing superalloys. Consequently, exploring new alloying elements is important. However, it has been difficult to explore new alloying elements having the same strengthening effect as Re by the existing methods [36–39].

**Figure 1.**
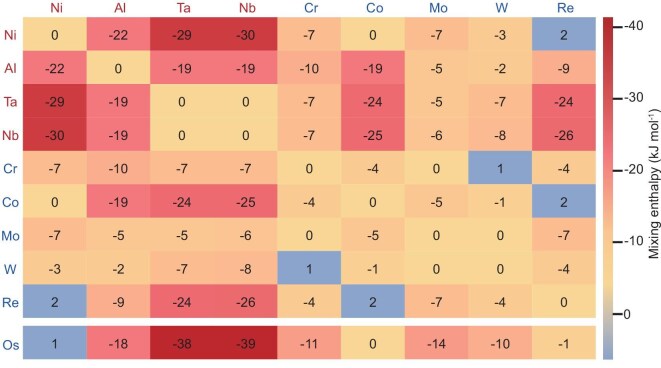
Comparison of the enthalpy values among different elements [44].

Creep resistance is determined primarily by the atomic bond energy for dislocation movements [38,39]. For example, recent studies have achieved enhanced resistance to dislocation movement through structural and composition design, thereby obtaining superalloys with excellent ultrahigh temperature creep properties [40,41]. Based on the strategy, the recent negative enthalpy (N-enthalpy < 0) design paradigm can be expected to provide some guidance [42,43]. In this study, mixing enthalpy design strategy is further developed to design Ni-based single crystalline superalloys. By following the N-enthalpy design rules, the large enthalpy value can increase the dislocation movement resistance by increasing the bond energy or the formation of local chemical ordering (LCO). By following the positive enthalpy (P-enthalpy ≥ 0) design rules, the positive value with Ni of the new element in γ phase can promote its segregation along the γʹ/γ interface, thus increasing the microstructural stability. By employing the combined N-enthalpy and P-enthalpy design strategies, the Os element is uniquely selected. Figure 1 illustrates a comparison of the enthalpy values between different elements [44]. The osmium (Os) element contains a P-enthalpy with Ni and an N-enthalpy with Al, Ta, Ti and Cr elements, which suggests that Os can be powerful for the strengthening effect.

Based on the mixing enthalpy alloying strategy, the Os mixing enthalpy alloying superalloy with excellent creep resistance is designed. Compared to the base superalloy, the creep properties under 1100°C/137 MPa, 980°C/250 MPa and 760°C/800 MPa conditions are comprehensively enhanced. The creep life of the Os-containing superalloys under 760°C/800 MPa conditions approaches 1273 h, more than almost six times that of the base alloy, setting a record among all existing metals and alloys. The cross-scale microstructural analysis indicates that the addition of the Os element introduced the interface effect, size effect and LCO. These findings provide novel insights into the design of Ni-based single crystalline superalloys with superior high-temperature performance.

## RESULTS AND DISCUSSION

### Microstructure and chemical distribution of the initial alloys

**Figure 2.**
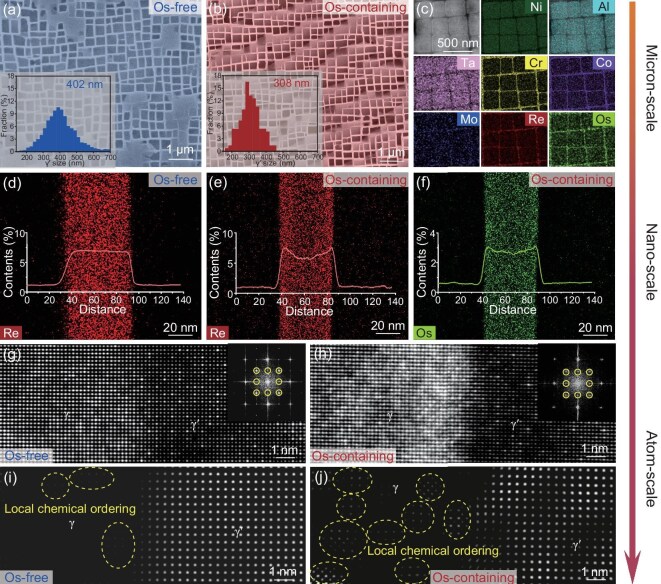
Initial microstructures of the two alloys, observed along the [001] direction. (a and b) γ′/γ morphologies of the Os-free and Os-containing alloys; the insets showing their γ′ phase size distribution. (c) EDS mapping image of the Os-containing alloy. (d) Distribution of the Re element across the γ′/γ interface in the Os-free alloy. (e and f) Distribution of the Re and Os elements across the γ′/γ interface in the Os-containing alloy. (g and h) Atomic-scale STEM-HAADF images of the Os-free and Os-containing alloys; the insets showing the FFT images. (i and j) IFFT images of the two alloys.

A comparison of the initial microstructure of the base (Os-free) and Os mixing enthalpy (Os-containing) alloys is shown in Fig. 2. Figure 2a shows the microscopic γʹ/γ morphology of the Os-free alloy. The cubic precipitate in dark contrast is the γʹ phase, whereas the surrounding matrix in bright contrast is the γ phase channel. The γ′ phase is an intermetallic compound with an L1_2_ structure, which primarily provides strength. The γ phase channel has a Ni-rich solid solution with a face-centred cubic structure, which primarily provides plasticity. As shown in the inset, the average γ′ phase cuboidal size is 402 nm and its volume fraction is about ∼69%. The average size of the γ phase channel is 90 nm.

Figure 2b shows the comparison results for the Os-containing alloy. It contains a γʹ/γ morphology similar to that of the Os-free alloys. However, the average size of its γ′ phase cubes is 308 nm, with a volume fraction of about ∼70%, and the average size of the γ channel is about 70 nm. Through the comparison of the size of γ and γ′ phases in the commercial superalloys reported in the literature, as shown in Fig. S2. It can be realized that the Os element is effective in decreasing the size of both γ′ phase and γ channel width.

The energy dispersive spectroscopy (EDS) mapping image of the Os-containing alloy is shown in Fig. 2c. Similar to the distribution in the Os-free alloy (as shown in Fig. S3), the Ni, Al and Ta elements segregated in the γ′ phase, whereas the Cr, cobalt (Co), Mo and Re elements segregated in the γ phase channel. The Os element exhibited the same distribution behaviour as Re, which segregated in the γ phase channel.

The reduction in the size of the γ′ phase caused by the addition of Os was affected primarily by the elements that were segregated at the interface. The distribution of the elements across the γ′/γ interface is shown in Fig. S4 and Fig. S5. Among these, there was no segregation of elements in the Os-free alloys, including the Re element, as shown in Fig. 2d. On the contrary, the segregation of Re at the γʹ/γ interface was evident in the Os-containing alloy. Moreover, the Os element was also segregated at the γʹ/γ interface, as shown in Fig. 2e and f, which was consistent with the P-enthalpy design rule. This would increase the stability of the interface, thus increasing the thermal stability and decreasing the size of the γ′ phase.

Figure 2g and h shows atomic-scale high-angle annular dark-field scanning transmission electron microscopy (STEM-HAADF) images of the two alloys. Some regions exhibit a higher contrast, indicating that a local cluster exists. The fast Fourier transform (FFT) pattern is shown in the inset. The inverse FFT (IFFT) images of the two alloys, using superlattice ordering analysis, are shown in Fig. 2i and j, respectively. The Os-free alloy exhibited limited LCO within the γ phase channels (as indicated by the yellow circle) while exhibiting long-range ordering in the γ′ phase. By comparison, the density of the LCO within the γ phase channels in the Os-containing alloy increased considerably. Additionally, the γ′ phase exhibited heterogeneous brightness variations, indicating the compositional fluctuations induced by the Os addition. These were consistent with the N-enthalpy design rule.

It can thus be realized that the addition of Os and other alloying elements introduces the N-enthalpy and P-enthalpy design strategies, which led to the formation of LCO and segregation at the γ/γ′ interface. The interface segregation reduced both the sizes of the γ′ phase and the γ channel by increasing the stability of the γ′ phase.

### Creep properties of the alloys

**Figure 3.**
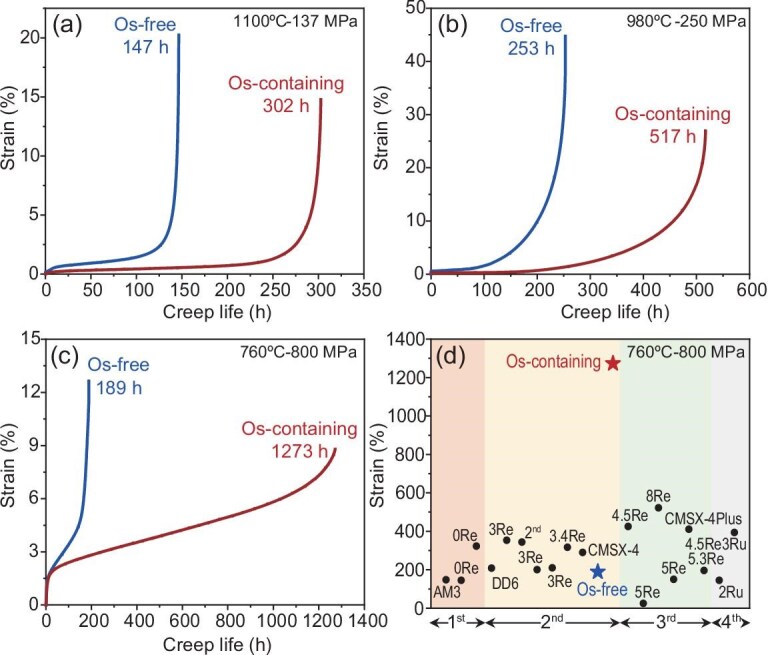
Creep properties of the Os-free and Os-containing superalloys under different conditions. (a–c) Creep curves of the two alloys tested under 1100°C/137 MPa, 980°C/250 MPa and 760°C/800 MPa conditions, respectively. (d) Comparison of the creep life tested under 760°C/800 MPa conditions of the current superalloys in the literature.

Figure 3 shows the creep properties of the Os-free and Os-containing superalloys under different conditions. Under high-temperature and low-stress conditions (1100°C/137 MPa), the creep life of the Os-free superalloy is 147 h, whereas that of the Os-containing alloy is 302 h, as shown in Fig. 3a. Under medium-temperature and medium-stress conditions (980°C/250 MPa), the creep life of the Os-free alloy is 253 h, whereas that of the Os-containing alloy is 517 h, as shown in Fig. 3b. Under medium-temperature and high-stress conditions (760°C/800 MPa), the creep life of the Os-free alloy is 189 h, whereas that of the Os-containing alloy is 1273 h, as shown in Fig. 3c. This indicates that the addition of Os comprehensively improved the creep life under different service conditions.

Table S2 shows the creep life tested under the above three conditions of typical superalloys in the literature. By comparison, the creep life tested under 760°C/800 MPa conditions reached a record high among all existing superalloys, as shown in Fig. 3d. The creep life tested under 1100°C/137 MPa and 980°C/250 MPa conditions also reached the highest level among all reported data in second-generation superalloys (Fig. S6).

### Strengthening effect caused by the microstructure

**Figure 4.**
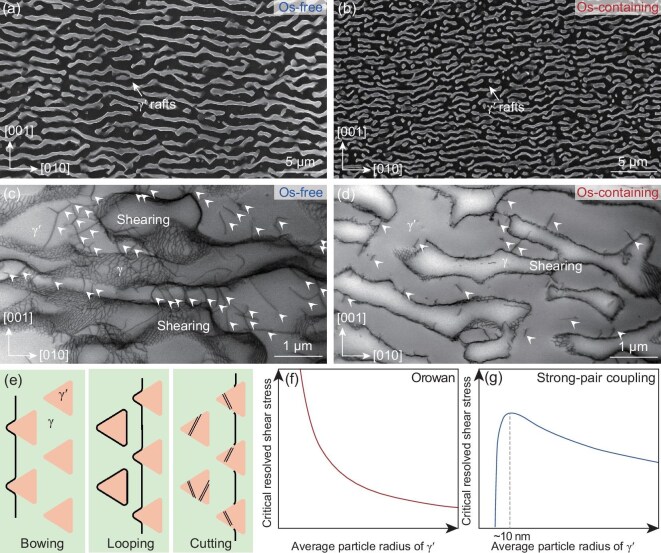
Microstructures of the Os-free and Os-containing alloys after creep under 980°C/250 MPa conditions. (a and b) SEM images observed along the [100] direction. (c and d) TEM images observed along the [100] direction. (e) Schematic image for the evolution of dislocations. (f and g) Critical resolved shear stresses for the dislocation movement mechanisms as functions of the average radius of the γ′ cuboids.

Based on the initial microstructure shown in Fig. 2, the addition of Os changed the microstructure at different scales. At the microscale, it reduced the size of the γ′ phase. At the nanoscale, it promoted the segregation of Re and Os at the γ′/γ interface. At the atomic scale, it increased the composition fluctuations, resulting in a high LCO density. Consequently, the strengthening effects caused by the microstructure could be divided into size effect, interface effect and LCO effect. Figure 4 shows the microstructure of the Os-free and Os-containing alloys after creep under the 980°C/250 MPa condition, to explore the influence of size on the creep resistance. Figure 4a shows a scanning electron microscopy (SEM) image of the Os-free alloy in the longitudinal direction. Clearly, the γ′ phase has completely drifted. Figure 4c shows a transmission electron microscopy (TEM) image of the Os-free alloy in the longitudinal direction. It is evident that the dislocations have sheared into the γ′ phase, as indicated by the arrows. Figure 4b and d show the same information for the Os-containing alloy. By comparison, the spacing of the raft structure and density of the shearing dislocation pairs in the γ′ phase were both smaller. This suggests that the addition of Os retards the coarsening of the γ′ phase. Its resulting reduction in γ′ size enhances the resistance to dislocation incision, thus promoting the alloys with higher creep resistance.

Figure 4e shows a schematic of the dislocation evolution. During the first stage, the dislocations are generated and move in the γ phase channels. When they are blocked by the γ′ phase, the Orowan process bypasses the γ′ blocks and enters the adjacent γ phase channels, leading to the accumulation of dislocations at the γ′/γ interfaces and the formation of dislocation networks. Subsequently, the accumulated dislocations begin to shear into the γ′ phase in the form of dislocation pairs. Throughout this process, the dislocation motion is affected by the Orowan resistance and strong pair shear. The Orowan resistance, as expressed in Eq. (1) [37], is inversely proportional to the size of the γ channel, i.e. the size of the γ′ phase, as shown in Fig. 4f. The smaller size of the γ and γ′ phases caused by the addition of Os thus increases the Orowan resistance to dislocation movement.

The critical resolved shear stress for strong pairing shear is calculated as in Eq. (2) [37]. As the size of the γ′ phase decreases, the resistance first increases and then decreases, with the size corresponding to the transition point being approximately 10 nm, as shown in Fig. 4g. Considering that the initial average size of the γ phase channels is greater than 10 nm, it can be deduced that a smaller γ′ phase size also increases the strong pair shear resistance. These two alloys contain almost the same volume fraction of the γ′ phase. Thus, the influence on the Orowan resistance and the strong pair shear resistance are mainly caused by the decrease of the sizes of the γ′ phase and γ channel. These two effects reduce the dislocation motion rate and dislocation density.

The addition of the Os element can create the size effect, which is an effective reinforcement mechanism against dislocation motion. It is worthwhile to notice that the morphology of the γ′ phase will transform from a cubical to a spherical shape when the size of γ′ phase further decreases [45,46]. It was demonstrated that some commercial alloys exhibit good creep resistance with the γ′ size of 300–500 nm, which implied that the γ′ phase in cubical morphology is effective in blocking the movement of the dislocations [47–62]. The ideal structure should consider the effects of both size and interface.

In addition, to give an insight into how the general tensile properties affect the creep behaviours, we conduct tensile tests at 760°C for both of the samples of Os-free and Os-containing samples, which are shown in Fig. S7. It can be realized that the yield strength of the Os-containing sample increases significantly by more than 200 MPa higher than the creep stress. It is thus implied that the alloy's higher yield strength at the service temperatures promotes the creep lives.


(1) \begin{eqnarray*}
{\tau }_{Or} = \sqrt {\frac{2}{3}} \frac{{Gb}}{{2{d}_{\gamma ^{\prime}}(f{\ }^{( - 1/3)} - 1)}},
\end{eqnarray*}

where ${\tau }_{Or}$ denotes the Orowan resistance, *G* denotes the shear modulus, *b* denotes the Burgers vector, *d_γ_*_′_ denotes the average size of the γ′ phase and *f* denotes the volume fraction of the γ′ phase.


(2) \begin{eqnarray*}
{\tau }_s\ = \ \sqrt {\frac{3}{2}} \left( {\frac{{Gb}}{{{d}_{\gamma ^{\prime}}}}} \right){f}^{1/2}\frac{\omega }{{{\pi }^{3/2}}}{\left( {\frac{{8\pi {E}_{APB}{d}_{\gamma ^{\prime}}}}{{\omega G{b}^2}} - 1} \right)}^{1/2},
\end{eqnarray*}

where ${\tau }_s$ denotes the critical resolved shear stress for strong pairing, *ω* denotes a dimensionless constant (usually 1) and *E_APB_* denotes the APB energy.

**Figure 5.**
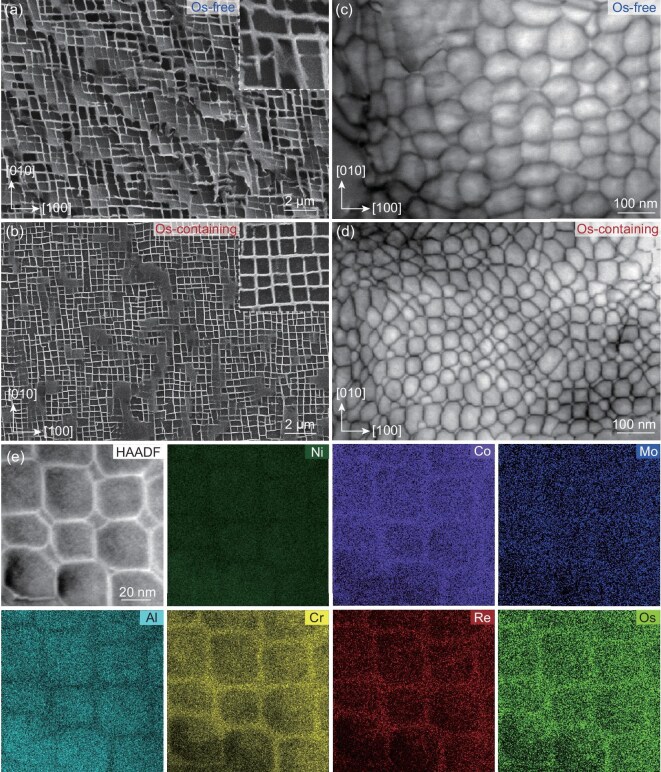
Microstructure of the Os-free and Os-containing alloys after creep under 760°C/800 MPa and 1100°C/137 MPa conditions. (a and b) SEM images observed along the [001] direction after creep under 760°C/800 MPa conditions. (c and d) TEM images observed along the [001] direction after creep under 1100°C/137 MPa conditions. (e) EDS mapping image of the dislocation network in the Os-containing alloy.

Figure 5 shows the microstructure of the Os-free and Os-containing alloys after creep under 760°C/800 MPa and 1100°C/137 MPa conditions, to explore the influence of the interface on the creep resistance. Figure 5a shows an SEM image of the Os-free alloy after testing under 760°C/800 MPa conditions. The γ′ phase remained cubic without any sign of rafting, except in some local regions where the γ′ cuboids may have become irregular in shape, as shown in the inset.

Figure 5b shows the same information for the Os-containing alloy. The cuboidal morphology of the γ′ phase remained practically unchanged, indicating that the Os addition enhanced the thermal stability of the γ′ phase. Figure 5c and d show the dislocation network in the two alloys after the creep test under the 1100°C/137 MPa condition. By comparison, the spacing of the dislocation network in the Os-free alloy was 60 nm, whereas that in the Os-containing alloy was ∼40 nm, indicating that the Os-containing alloy exhibited a larger degree of lattice misfit, which was consistent with the measured misfit shown in Fig. S8. This resulted in higher back stress for dislocation motion, thereby promoting higher creep resistance.

The EDS mapping of the dislocation network is shown in Fig. 5e. Besides the Cr, Co and Re elements, the Os element was also enriched at the dislocations. The segregation of these elements (typically the low diffusion elements Re and Os) slowed the dislocation movement by increasing its slip and climb resistance. This, in turn, improved the thermal stability of the γ′/γ interface.

Apart from the size effect, the interfacial effect is another critical factor to consider in microstructure design. The segregation of key alloying elements at the interfaces can enhance the thermal stability of the interface. This may lead to a lower coarsening rate of the γ′ phase, along with reduced mobility of dislocation movement, thereby decreasing the creep rate. Consequently, the synergistic effects of both size and interface effects should be incorporated during alloy design to enhance the overall creep resistance of the alloy.

**Figure 6.**
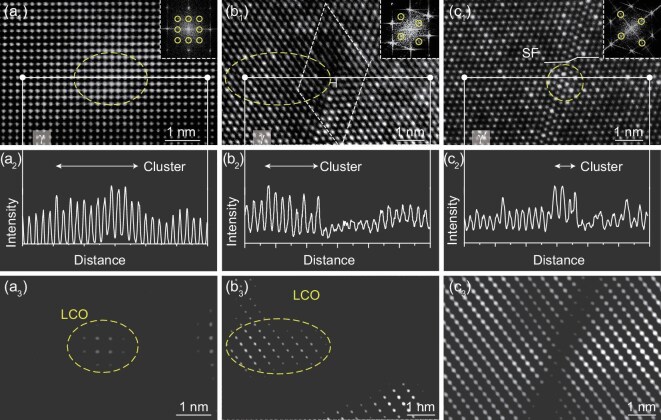
Mechanism of cluster interaction with defects. (a_1_) Cluster in the γ phase channels. (a_2_ and a_3_) Atomic intensities lining analyses and IFFT image of (a_1_). (b_1_) LCO interaction with the dislocations in the γ phase channels. (b_2_ and b_3_) Atomic intensities lining analyses and IFFT image of (b_1_). (c_1_) Segregation interaction with the stacking fault in the γ′ phase. (c_2_ and c_3_) Atomic intensities lining analyses and IFFT image of (c_1_).

The mechanism of the cluster interaction with defects is shown in Fig. 6. Figure 6a1 shows the HAADF image of the cluster in the γ phase channels. The variation in *Z* contrast indicates the presence of local heavy-element separation. The atomic intensity lining analysis and IFFT image of Fig. 6a1 are shown in Fig. 6a2 and 6a3. They indicate that LCO existed in the cluster formation region. Figure 6b1–3 show the LCO interaction with the dislocations in the γ phase channels. It is evident that the dislocation pores were pinned by the LCO structure, which considerably increased the critical resolved shear stress for dislocation slip, thereby increasing the creep resistance. Figure 6c1–3 show the interaction of the cluster with the stacking fault in the γ′ phase. The cluster was shown to be Cr, Co, Mo, Re and Os segregation, as indicated in Fig. S9. This configuration considerably enhanced the resistance to stacking-fault movement, which also increased the creep resistance.

## CONCLUSIONS

In conclusion, the negative mixing enthalpy and mixing enthalpy alloying using Os largely optimized the microstructures of Ni-based single crystalline alloys. The highlighted P-enthalpy alloying strategy using Os leads to an interface effect via segregation of the Os and Re elements on the γ/γ′ interface. The N-enthalpy alloying strategy using Os leads to LCO formation with Cr and others in the γ phase channels. The segregation of Os and Re further produces the size effect by confining the growth of the γ′ phase to a smaller size with smaller γ channels, which, in turn, creates small-size resistance effects for dislocation nucleation and movements based on Hall–Petch rules. The LCO and clusters in the γ phase slowed the dislocation movement and the combined effects increased the microstructural stability during creep. Compared with the base alloy, the creep life of the mixing enthalpy Os alloying superalloy under 1100°C/137 MPa, 980°C/250 MPa and 760°C/800 MPa conditions is comprehensively improved. Moreover, the creep life tested under 760°C/800 MPa conditions reached a record high of 1273 h among all existing metals and alloys. This study sheds new light on the route of materials design through interface engineering and size effects by mixing enthalpy strategy and N-enthalpy alloying, which promises a new paradigm for designing metals and alloys with excellent high-temperature mechanical performance.

## METHODS

### Material preparation

Two different Ni-based single crystalline superalloys were used in this study. The nominal chemical compositions of the two alloys are given in Table S1. The base and Os mixing enthalpy alloying (Os-containing) alloys differed only in Os content and are thus denoted as ‘Os-free’ and ‘Os-containing’ in this article. The Ni-based superalloy single crystalline cylindrical bars were fabricated using directional solidification investment casting. They measured Φ16 mm × 220 mm with the [001] orientation along the bar axial direction. The orientation deviation was measured using electron backscatter diffraction (EBSD), as shown in Fig. S1. Both were located adjacent to the [001] direction, and the margin of orientation variation was estimated to be within 2°. Solution and two-step ageing heat treatments were applied as follows: 1290°C/1 h + 1300°C/2 h + 1315°C/4 h + 1120°C/4 h + 870°C/32 h (air cooling, AC) for the Os-free alloy and 1290°C/1 h + 1300°C/2 h + 1315°C/4 h + 1320°C/4 h + 1325°C/4 h + 1120°C/4 h + 870°C/32 h (AC) for the Os-containing alloy. After heat treatment, the samples were fabricated into dumbbell shapes with a cross-sectional dimension of ϕ5 mm × 27 mm along the [001] axis direction. Creep testing was performed under 1100°C/137 MPa, 980°C/250 MPa and 760°C/800 MPa conditions.

### Microstructural analysis

X-ray diffraction (XRD), SEM and TEM were used to analyse the microstructures. Specimens for the SEM and TEM studies were prepared using the standard metallography process to obtain high-quality surfaces. The SEM samples were chemically etched using a solution of 40 g of CuSO_4_, 160 mL of HCl and 200 mL of H_2_O. SEM examination was conducted using an FEI Quanta 650F microscope. The TEM specimens were prepared using twin-jet electrochemical polishing in an electrolyte solution containing 5% HClO_4_ and 95% C_2_H_5_OH. TEM examination was conducted using an ETEM Cs-corrected 300 kV analytical transmission electron microscope. STEM-mode HAADF images and EDS analysis were adopted. The XRD spectra were obtained using a D8 Advance-X-ray diffractometer with a Cu Kα X-ray source. Image analysis was conducted using Image-Pro Plus software to measure the size of the γ′ phase.

## Supplementary Material

nwaf228_Supplemental_File
