# Supplementary material for: Negative mixing enthalpy and mixing enthalpy alloying leads to interface and size effects towards superb creep resistance of nickel-based single crystalline superalloys
Source: Natl Sci Rev. 2025 Jun 9;12(8):nwaf228. doi: 10.1093/nsr/nwaf228 (PMC12236342; doi:10.1093/nsr/nwaf228)
Supplement: nwaf228_Supplemental_File [file nwaf228_supplemental_file.pdf]

Supplementary materials

Negative mixing enthalpy and mixing enthalpy alloying leads to interface and size effects towards superb creep resistance of nickel-based single crystalline superalloys

Junbo Zhao[1#], Xiaoyi Yuan[1#], Yunsong Zhao[2#], Zhanxin Wang[1], Haibo Long[1,*], Shengcheng Mao[1], Lihua Wang[1], Xiaodong Han[1,3,*] and Ze Zhang[4,*]

[1]Beijing Key Laboratory of Microstructure and Property of Advanced Materials, College of Materials Science and Engineering, Beijing University of Technology, Beijing, 100124, China

[2]Science and Technology on Advanced High Temperature Structural Materials Laboratory, Beijing Institute of Aeronautical Materials, Beijing, 100095, China

[3]Department of Materials Science & Engineering, Southern University of Science and Technology, Shenzhen, 518000, China

[4]State Key Laboratory of Silicon Materials and Department of Materials Science and Engineering, Zhejiang University, 310058 Hangzhou, China

[#] These authors contributed equally

* Corresponding author email: hblong@bjut.edu.cn (H. Long), xdhan@bjut.edu.cn (X. Han), zezhang@zju.edu.cn (Z. Zhang)

Contents

Supplementary Table 1 Nominal composition of the experimental alloys 3

Supplementary Table 2 Comparison of creep performance under different conditions 3

Supplementary Figure 1 EBSD orientation analysis of the two alloys. 4

Supplementary Figure 2 The comparison of the γ and γ′ phases' size between different alloys in the literature 4

Supplementary Figure 3 EDS mapping image of the Os-containing alloy. 5

Supplementary Figure 4 Distribution of elements across the γ′-γ interface of the Os-free alloy. 6

Supplementary Figure 5 Distribution of elements across the γ′-γ interface of the Os-containing alloy. 7

Supplementary Figure 6 Comparison of the creep life with the second-generation superalloys under different test conditions 8

Supplementary Figure 7 Tensile stress-strain curves of alloys at 760°C 8

Supplementary Figure 8 XRD patterns of the Os-free and Os-containing alloys...9

Supplementary Figure 9 Distribution of elements in the laminar faults of the Os-containing alloys. 9

Supplementary Table 1 Nominal composition of the experimental alloys (wt.%)

Name	Ni	Al	Ta	Nb	Cr	Co	Mo	W	Re	Os	Hf
Os-free	Bal.	5.6	7.5	0.5	4.3	9.0	2.0	8.0	2.0	0	0.1
Os-containing	Bal.	5.6	7.5	0.5	4.3	9.0	2.0	8.0	2.0	1.0	0.1

Supplementary Table 2 Comparison of creep performance under different conditions

Type	Name	Life(h)	Name	Life(h)	Name	Life(h)
1100℃-137MPa	DD6	146[1]	2Re	117[2]	TMS-285	195[3]
	3Re	194[4]	DD6	206[2]	TMS-244	190[5]
	Rene N5	89[6]	2.9Re	122[7]	2.85Re	184.5[8]
	CMSX-4	139[6]	DD6	148[9]	3Re	218[10]
	2Re	132[11]	TMS-286	147[3]		
980℃/250MPa	DD6	274[12]	2Re	229[13]	2Re	260[14]
	PWA1484	218[11]	2.5Re	226[15]	3Re	290[16]
	Rene N5	228[11]	2.5Re	240[15]	CMSX-4	230[17]
	2Re	234[18]	2.5Re	217[15]	CMSX-4M	314[17]
	CMSX-4	178[11]	2.5Re	189[15]	2Re	190[19]
	2Re	254[20]	2Re	316[21]	2Re	259[22]
	3Re	225[23]	3Re	239[24]	3Re	330[24]
	AM3	148[1]	3Re	200[10]	8Re	522[25]
760℃/800MPa	0Re	145[26]	3Re	210[27]	5Re	150[28]
	0Re	323[29]	3.4Re	317[27]	CMSX-4P	411[27]
	DD6	208[20]	CMSX-4	290[27]	5.3Re	196[27]
	3Re	353[30]	4.5Re	425[31]	2Ru	145[32]
	2nd	344[33]	5Re	25[34]	4.5Re3Ru	394[35]

.

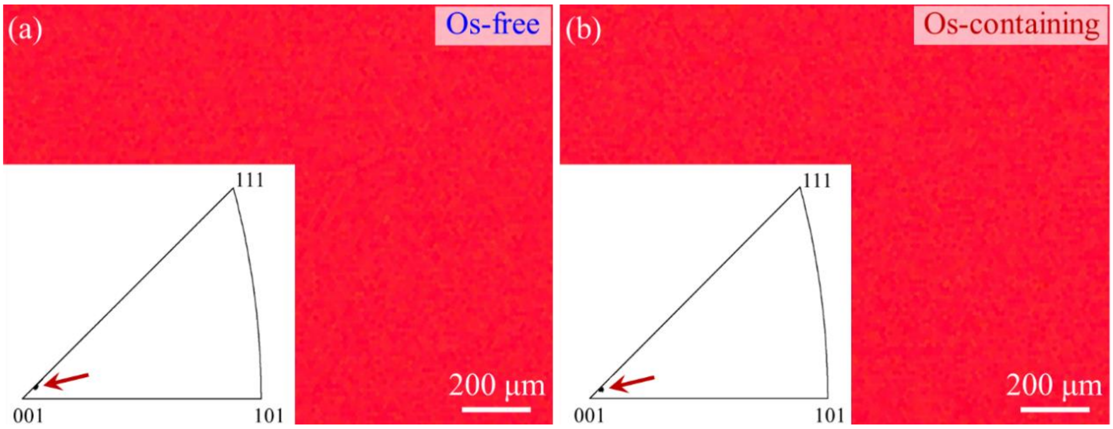

Supplementary Figure 1 EBSD orientation analysis of the two alloys. (a) Orientation image of the Os-free alloy in the axial direction. (b) Orientation image of the Os-containing alloy in the axial direction. The insets are the inverse pole figures for the two alloys.

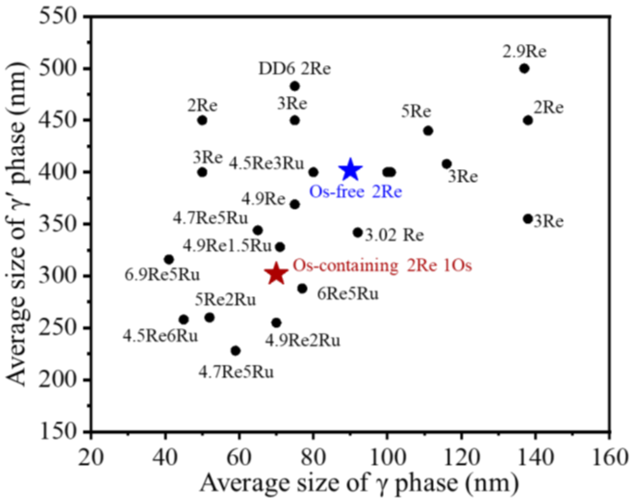

Supplementary Figure 2 The comparison of the γ and γ′ phases' size between different alloys in the literature [2, 4, 6, 7, 10, 17, 19, 20, 22-25, 31, 35-39].

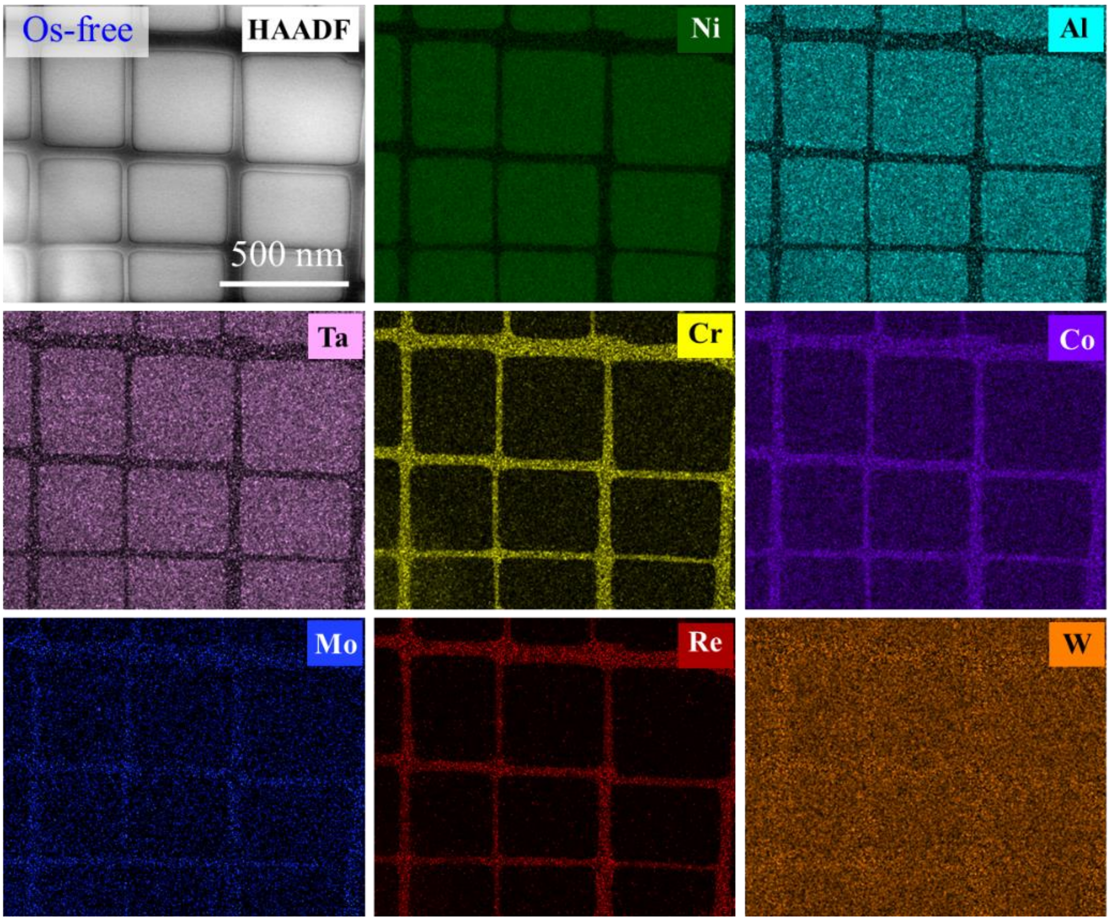

Supplementary Figure 3 EDS mapping image of the Os-containing alloy**.**

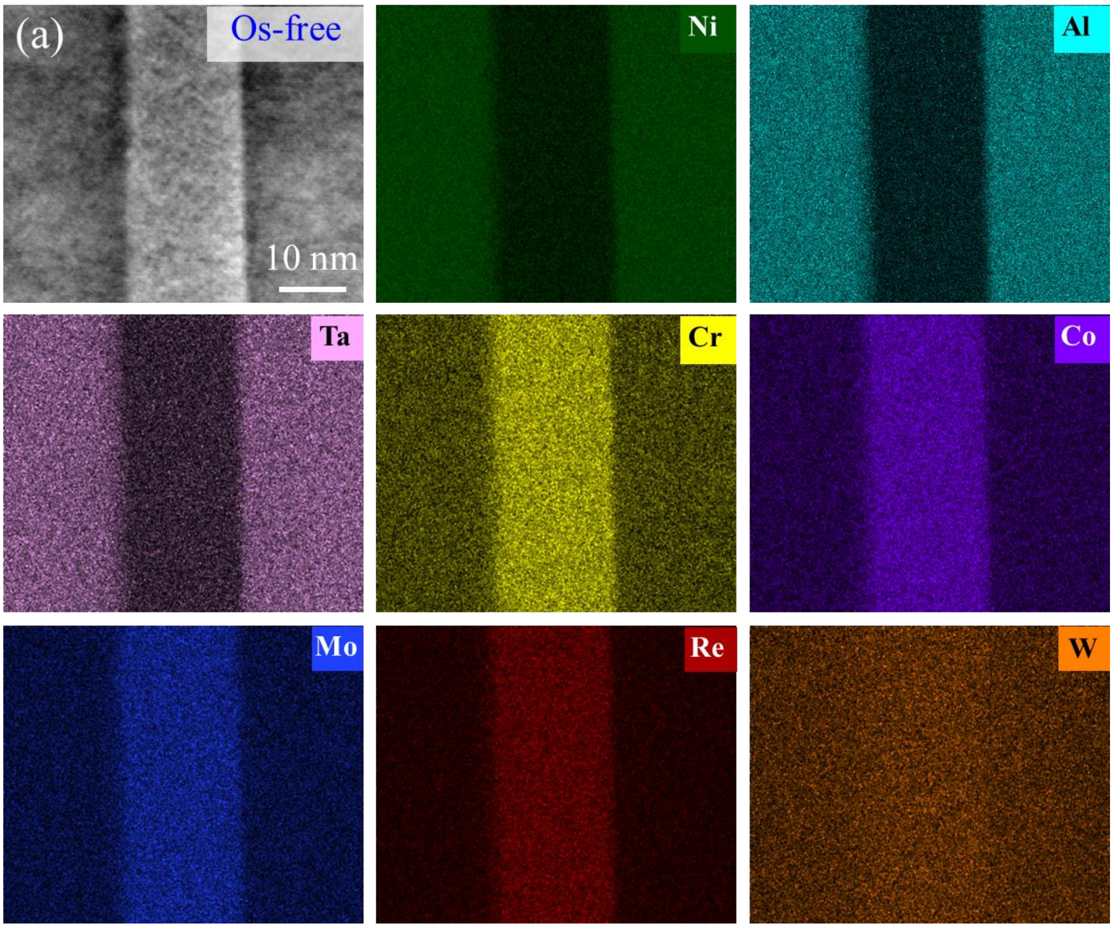

Supplementary Figure 4 Distribution of elements across the γ′-γ interface of the Os-free alloy.

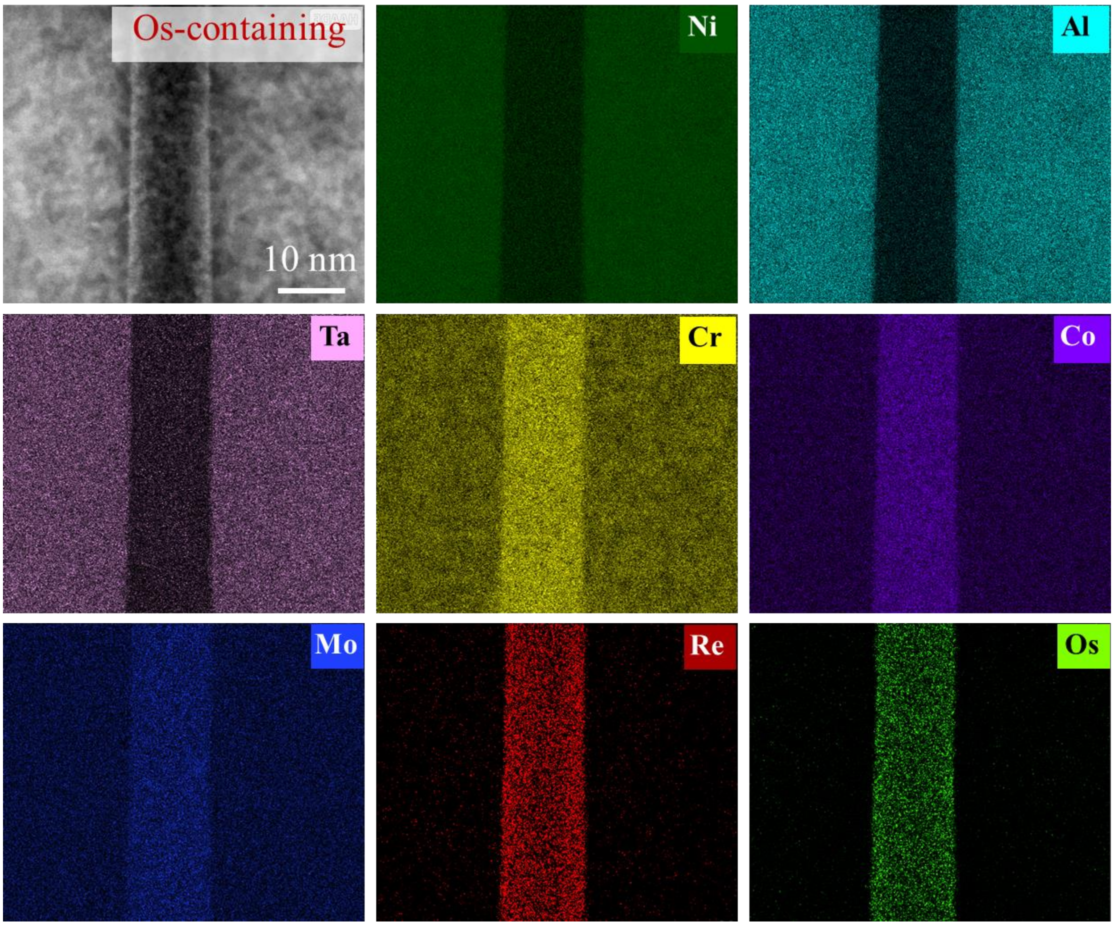

Supplementary Figure 5 Distribution of elements across the γ′-γ interface of the Os-containing alloy.

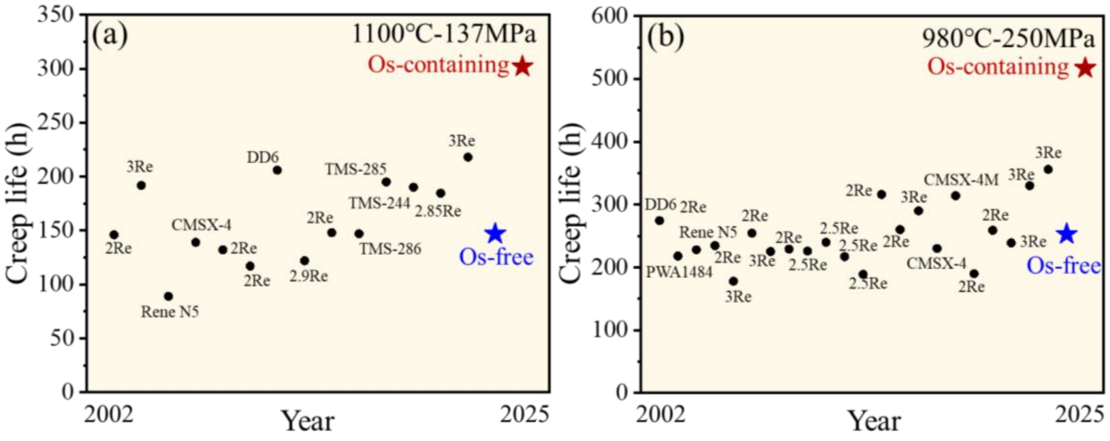

Supplementary Figure 6 Comparison of the creep life with the second-generation superalloys under different test conditions [1, 10-35]. (a)1100°C–137 MPa. (b) 980°C–250 MPa.

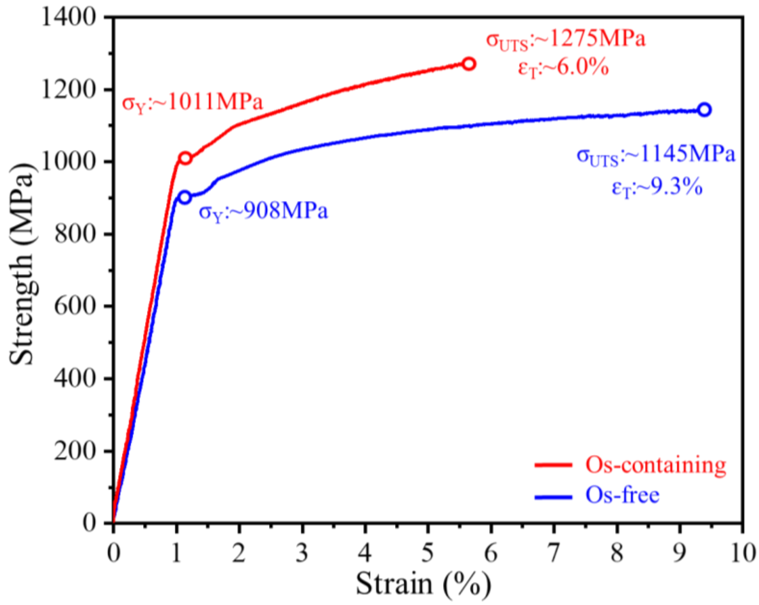

Supplementary Figure 7 Tensile stress-strain curves of alloys at 760°C

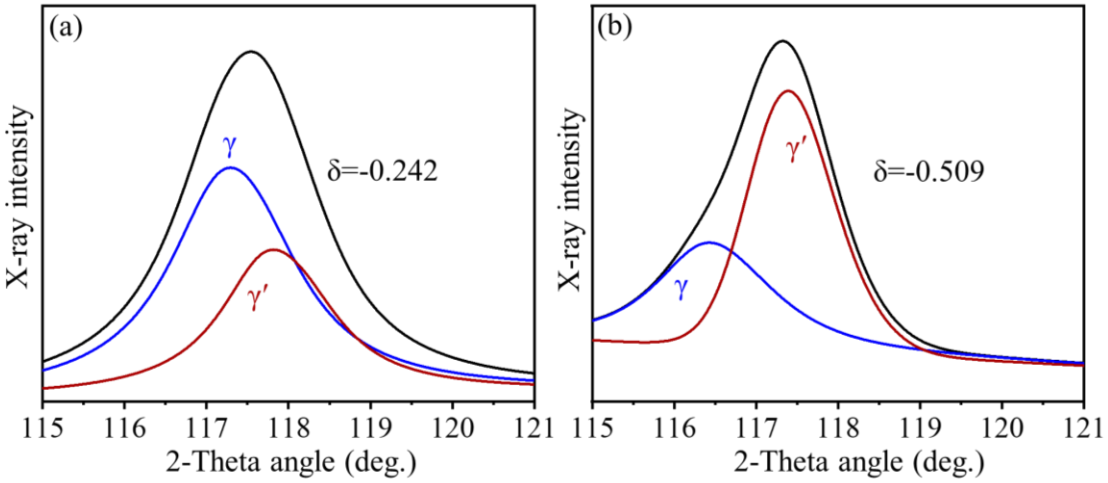

Supplementary Figure 8 XRD patterns of the Os-free and Os-containing alloys. (a) Os-free alloy. (b) Os-containing alloy.

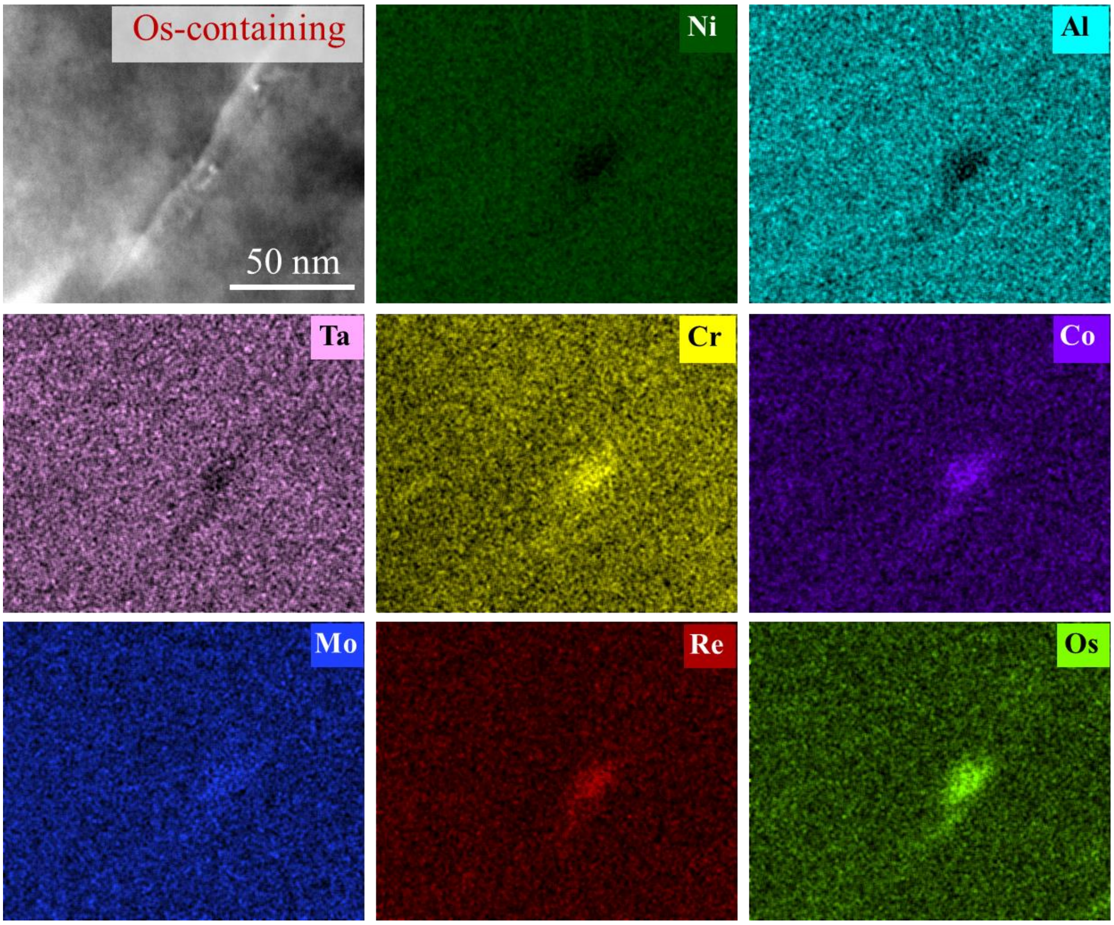

Supplementary Figure 9 Distribution of elements in the stacking-faults of the Os-containing alloys.

Reference

1. Academic Committee of the Superalloy C. *China Superalloys Handbook. Beijing*: Standards Press of China, 2008, 549-64.
2. Sun N, Zhang L, Li Z *et al.* The effect of microstructure on the creep behavior of a low rhenium-containing single crystal nickel-based superalloy. *Mater Sci Eng A* 2014; **606**: 175-86.
3. Kawagishi K, Yokokawa T, Kobayashi T *et al.* Development of low or zero-rhenium high-performance Ni-base single crystal superalloys for jet engine and power generation applications. *Superalloy 2016* 2016: 115-22.
4. Ma W, Han Y, Li S *et al.* Effect of Mo content on the microstructure and stress rupture of a ni base single crystal superalloy. *Acta Metall Sin* 2006; **11**: 1191-96.
5. Koizumi Y, Kawagishi K, Yokokawa T *et al.* Hot corrosion and creep properties of Ni-base single-crystal superalloys. *Superalloy 2020* 2020: 747-52.
6. Koizumi Y, Yokokawa T, Harada H *et al.* Database of creep property for nickle-base single crystal superalloys, Rene N4, Rene N5 and CMSX-4. *J Jpn Inst Met Mater* 2006; **70**: 176-79.
7. Xiang S, Mao S, Wei H *et al.* Selective evolution of secondary γ′ precipitation in a Ni-based single crystal superalloy both in the γ matrix and at the dislocation nodes. *Acta Mater* 2016; **116**: 343-53.
8. Huang J, Ai C, Ru Y *et al.* The effect of cooling rate from solution treatment on γ′ reprecipitates and creep behaviors of a Ni-based superalloy single-crystal casting. *Crystals* 2022; **12**: 1235-55.
9. Li J, Liu S, Wang X *et al.* Development of a low-cost third generation single crystal superalloy DD9. *Superalloy 2016* 2016: 55-63.
10. Tan Z, Wang X, Du Y *et al.* Microstructural stability and creep performance of a novel low-cost single crystal superalloy. *Met Mater Int* 2022; **28**: 1599-610.
11. Li J, Liu S, Shi Z *et al.* Third generation single crystal superalloy DD9. *J Iron Steel Res Int* 2011; **23**: 337-40.
12. Li J, Sun F, Xiong J *et al.* Effects of surface recrystallization on the microstructures and creep properties of single crystal superalloy DD6. *Mater Sci Forum* 2010; **638**: 2279-84.
13. Hu Y, Zhang L, Cao T *et al.* The effect of thickness on the creep properties of a single-crystal nickel-based superalloy. *Mater Sci Eng A* 2018; **728**: 124-32.
14. Huang Y. Effect of Heat Treatment System on Microstructure and Properties of DD6 Nickel-Based Single Crystal Superalloy. *Masteral Thesis.* Harbin Engineering University, 2020.
15. Shi Z, Liu S, Zhao J. Effect of C content on microstructures and stress rupture properties of a single crystal superalloy. *Nonferrous Met Mater Eng* 2018; **39**: 1-6.
16. Wu R, Zhao Y, Liu Y *et al.* High temperature creep mechanisms of a single crystal superalloy: a phase-field simulation and microstructure characterization. *Prog Nat Sci* 2020; **30**: 366-70.
17. Xuan W, Song G, Duan F *et al.* Enhanced creep properties of nickel-base single crystal superalloy CMSX-4 by high magnetic field. *Mater Sci Eng A* 2021; **803**: 140729.
18. Shi Z, Li J, Liu S *et al.* Effects of dendritic orientation on stress rupture properties

of DD6 single crystal superalloy. *J Iron Steel Res Int* 2011; **18**: 66-71.
19. Liang J, Wang J, Zhang D *et al.* Coupling model for the gradient loading creep behavior of nickel-based single-crystal superalloys. *Eng Fract Mech* 2023; **294**: 109688.
20. Fan Y, Shi H, Qiu W. Constitutive modeling of creep behavior in single crystal superalloys: effects of rafting at high temperatures. *Mater Sci Eng A* 2015; **644**: 225-33.
21. Zhang L, Yu H, Guo G *et al.* Stress rupture properties and fracture behavior of thin wall specimens of DD6 single crystal superalloy with [001] direction. *J Aero Power* 2019; **34**: 627-34.
22. Yu J, Li J, Fang X *et al.* Influence of secondary γ′ phase evolution on creep properties of single crystal superalloy DD6. *J Mater Eng* 2023; **51**: 60-6.
23. Liu S, Shi Z, Xiong J *et al.* Microstructure and stress rupture properties of a single crystal superalloy. *Nonferrous Met Mater Eng* 2017; **8**: 118-21.
24. Song Y, Fan J, Li J *et al.* New insights into the optimisation of the solution heat treatment process and properties of CMSX-4 superalloys. *Mater Sci Eng A* 2024; **890**: 145947.
25. Gu S, Gao H, Wen Z *et al.* Creep properties and life model of anisotropic Ni-based single crystal superalloys over a wide temperature range. *Int J Mech Sci* 2024; **261**: 108674.
26. Tian S, Shu D, Zeng Z *et al*. influence of element Re on intermediate temperature creep behavior of single crystal nickle-base superalloy. *Trans Mater Heat Treat* 2013; **34**: 17-54.
27. Ormastroni L, Rame J, Cormier J. Creep properties dependence to solution heat treatment of second and third generation Ni-based single crystal superalloys. *Superalloys 2024* 2024: 473-83.
28. Liu J, Sun J, Meng J *et al.* Microstructural stability and stress rupture properties of a third-generation Ni base single crystal superalloy. *Acta Metall Sin* 2024; **60**: 770-6.
29. Tian S, Ding X, Guo Z *et al.* Damage and fracture mechanism of a nickel-based single crystal superalloy during creep at moderate temperature. *Mater Sci Eng A* 2014; **594**: 7-16.
30. Liu S, Shi Z, Xiong J *et al.* Microstructure and stress rupture properties of a single crystal superalloy. *Nonferrous Metals Science and Engineering*. 2017; **8**: 118-21.
31. Tian S, Zeng Z, Zhang C *et al.* Creep behavior and its effect factors of a single crystal nickel-based superalloy containing 4.5%re at medium temperature. *Rare Metal Mat Eng* 2013; **42**: 494-99.
32. Liang S, Liu Z, Fu Y *et al.* Research on creep behaviors of a single crystal nickel-base superalloy containing 2% Ru. *Heat Treat* 2016; **31**: 13-8.
33. Chen S. Effect of sulfur on the microstructure and mechanical properties of nickel-based single crystal superalloy. *Masteral Thesis*, Shenyang University of Technology, 2021.
34. Sun J, Liu J, Chen C *et al.* Effect of γ′ size on intermediate temperature stress rupture property of the third generation single crystal nickel-base superalloy containing re. *Rare Metal Mat Eng* 2022; **51**: 369-73.
35. Shu D, Tian S, Liu L et al. Elements distribution and deformation features of a 4.5%

Re nickel-based single crystal superalloy during creep at high temperature. *Mater Charact* 2018; **141**: 433-41.
36. Zhang J, Wang J, Harada H *et al.* The effect of lattice misfit on the dislocation motion in superalloys during high-temperature low-stress creep. *Acta Mater* 2005; **53**: 4623-33.
37. Toshiharu K, Hiroshi H, Makoto O *et al.* Creep strengths of Ir-containing 5th generation SC superalloys. *J Jpn Inst Met Mater* 2005; **69**: 1099-103.
38. Koizumi Y, Kobayashi T, Zhang J *et al.* Development of next-generation Ni-base single crystal superalloys. *Superalloys 2004* 2004: 35-43.
39. Long H, Zhao Y, Zhao J *et al.* Superb creep lives of Ni-based single crystal superalloy through size effects and strengthening heterostructure γ/γ′ interfaces. *Natl Sci Open* 2024; **3**: 20230042.
